# Exploiting Wild Relatives for Genomics-assisted Breeding of Perennial Crops

**DOI:** 10.3389/fpls.2017.00460

**Published:** 2017-04-04

**Authors:** Zoë Migicovsky, Sean Myles

**Affiliations:** Department of Plant, Food and Environmental Sciences, Faculty of Agriculture, Dalhousie University,Truro, NS, Canada

**Keywords:** crop wild relatives, perennials, marker-assisted selection, association mapping, linkage mapping, genomics-assisted breeding

## Abstract

Perennial crops are vital contributors to global food production and nutrition. However, the breeding of new perennial crops is an expensive and time-consuming process due to the large size and lengthy juvenile phase of many species. Genomics provides a valuable tool for improving the efficiency of breeding by allowing progeny possessing a trait of interest to be selected at the seed or seedling stage through marker-assisted selection (MAS). The benefits of MAS to a breeder are greatest when the targeted species takes a long time to reach maturity and is expensive to grow and maintain. Thus, MAS holds particular promise in perennials since they are often costly and time-consuming to grow to maturity and evaluate. Well-characterized germplasm that breeders can tap into for improving perennials is often limited in genetic diversity. Wild relatives are a largely untapped source of desirable traits including disease resistance, fruit quality, and rootstock characteristics. This review focuses on the use of genomics-assisted breeding in perennials, especially as it relates to the introgression of useful traits from wild relatives. The identification of genetic markers predictive of beneficial phenotypes derived from wild relatives is hampered by genomic tools designed for domesticated species that are often ill-suited for use in wild relatives. There is therefore an urgent need for better genomic resources from wild relatives. A further barrier to exploiting wild diversity through genomics is the phenotyping bottleneck: well-powered genetic mapping requires accurate and cost-effective characterization of large collections of diverse wild germplasm. While genomics will always be used in combination with traditional breeding methods, it is a powerful tool for accelerating the speed and reducing the costs of breeding while harvesting the potential of wild relatives for improving perennial crops.

## Introduction

Perennials, or species that live for more than 2 years, include herbaceous plants, woody shrubs, and trees (Miller and Gross, 2011). Although most agriculturally important crops are annuals, perennials occupy over 13% of the world’s surface area dedicated to food production (**Table 1**) (Food and Agriculture Organization of the United Nations, 2017). Not only are perennial crops a vital contributor to global food production and nutrition, but many offer advantages over annual crops. For example, perennial species generally have longer growing seasons (Dohleman and Long, 2009), increased root carbon (Glover et al., 2010a), and reduced soil erosion risk (Vallebona et al., 2016) when compared to annuals. As a result, there is increasing interest in perennializing annual grains (Glover et al., 2010b; Kane et al., 2016). While there are many benefits to growing perennials, breeding new cultivars is expensive and time-consuming due to the large size and lengthy juvenile phase of many species. For example, an avocado tree (*Persea americana*) may take up to 15 years to mature before flowering (Berg and Lahav, 1996). The recent breeding of 3 commercial apple (*Malus domestica*) cultivars took 26 years (Peil et al., 2008), and thus, it is common for a limited number of elite cultivars to be propagated widely for long periods of time. For example, the ‘McIntosh’ apple is over 200 years old, while the ‘Pinot Noir’ grape (*Vitis vinifera*) has been grown for a millennium. Propagation of the same cultivars for decades—if not centuries—results in increasing susceptibility to disease, since these crops remain genetically frozen while pathogens continue to evolve (Myles, 2013). Over 75% of perennial crops are vegetatively propagated and the extensive use of a small number of elite cultivars fails to exploit the immense phenotypic and genetic diversity available (Miller and Gross, 2011). Expanding the breeding pool to include wild relatives can provide a crucial new source of desirable traits for introgression into perennial crops.

**Table 1:** The top 20 perennial crops based on total global area.

Crop	Global area (million hectares)	Global contribution (%)	Perennial contribution (%)
Sugar cane (*Saccharum* spp.)	27.1	2.03	15.2
Palm fruit (*Elaeis* spp.)	18.7	1.4	10.5
Coconuts (*Cocos nucifera*)	11.9	0.894	6.71
Rubber (*Hevea brasiliensis*)	11.1	0.831	6.24
Coffee (*Coffea arabica*)	10.5	0.785	5.89
Cocoa (*Theobroma cacao*)	10.4	0.781	5.87
Olives (*Olea europaea*)	10.3	0.769	5.77
Grapes (*Vitis vinifera*)	7.12	0.534	4
Pigeon peas (*Cajanus cajan*)	7.03	0.527	3.95
Cashew (*Anacardium occidentale*)	6.04	0.452	3.39
Mangoes (*Mangifera indica*), mangosteens (*Garcinia mangostana*), guavas (*Psidium guajava*)	5.64	0.423	3.17
Bananas (*Musa* spp.)	5.39	0.404	3.03
Apples (*Malus domestica*)	5.05	0.378	2.84
Plantains (*Musa* spp.)	4.5	0.337	2.53
Oranges (*Citrus* spp.)	3.89	0.291	2.18
Tea (*Camellia* spp.)	3.8	0.285	2.14
Plums, sloes (*Prunus* spp.)	2.52	0.189	1.42
Tangerines, mandarins, clementines, satsumas (*Citrus* spp.)	2.28	0.171	1.28
Almonds (*Prunus dulcis*)	1.73	0.13	0.974
Pears (*Pyrus communis*)	1.57	0.118	0.885

*Total global area, in million hectares, is listed as well as the proportion of total area (annuals and perennials) and proportion of perennial area for each of these crops. The total global area for all crops is estimated at 1335.37 million hectares, while the global area for perennial crops is estimated at 177.90 million hectares. Values are calculated based on the most recent available year of data (2014) from the Food and Agriculture Organization of the United Nations (2017) website (http://www.fao.org/faostat). Crops we were unable to categorize due to ambiguous names which included both perennial and annual species were excluded.*

Crop wild relatives (CWRs) provide an invaluable resource for improving perennial crops through disease resistance, fruit quality, and rootstocks. By 1997, improvements to crops due to CWRs had an estimated global benefit of $115 billion annually (Pimentel et al., 1997). However, the definition of what constitutes a ‘CWR’ can be unclear, especially in perennial species where only a few generations of breeding may have occurred since domestication. For example, in kiwifruit (*Actinidia* spp.) almost all cultivars were either taken directly from the wild or are the result of only two-to-three generations of breeding. Commercial kiwifruit cultivars, including ‘Hayward’ (*Actinidia chinensis* var. *deliciosa*), the most widely grown cultivar, are very similar to wild plants (Ferguson, 2007; Ferguson and Huang, 2007). Likewise, cranberry (*Vaccinium macrocarpon*) cultivars are generally either wild selections or only a few generations removed (Fajardo et al., 2012). Finally, most banana cultivars (*Musa* spp.) are vegetatively propagated wild individuals collected by farmers due to the presence of parthenocarpic fruit which develop without seeds, pollination, or fertilization (Heslop-Harrison and Schwarzacher, 2007). When many elite perennial cultivars are in fact simply wild plants selected for cultivation with minimal improvement or domestication, the concept of CWRs becomes blurred.

**FIGURE 1:**
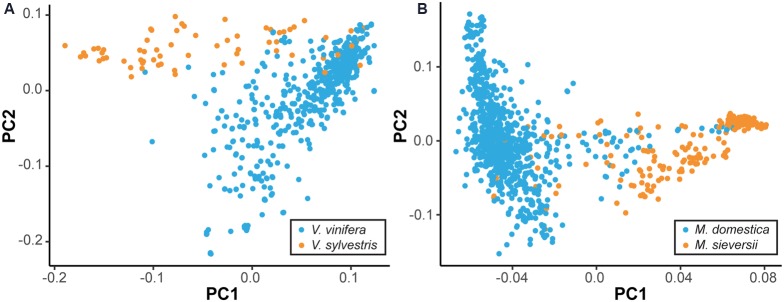
**Lack of differentiation between wild and domesticated perennial species.** By plotting the two major axes of variation against each other (i.e., PC1 vs. PC2) we gain an overview of the genetic relatedness among samples. The primary wild ancestors and domesticated species cannot be clearly separated. Principal component analysis (PCA) was performed using single nucleotide polymorphism (SNP) data to compare primary progenitor species and cultivated accessions of grape **(A)** and apple **(B)**. Cultivated accessions, as labeled by the USDA, are indicated in blue, while the primary progenitor species are indicated in orange. Equal sample sizes were used for both species and additional samples were projected onto the PCA axes.

While many cultivated perennial crops are essentially wild, even crops that have been bred for millennia are often not genetically distinct from their wild ancestors. To demonstrate this, we used genome-wide single nucleotide polymorphism (SNP) data to compare the primary progenitor and cultivated species of grape (*Vitis*) and apple (*Malus*) using principal component analysis (PCA) (**Figure 1**) (Myles et al., 2011, Gardner et al., unpublished). **Figure 1** suggests no clear differentiation between the domesticated *V. vinifera* and the wild progenitor *Vitis sylvestris*, and the same is true of the domesticated *M. domestica* and its primary progenitor species *Malus sieversii*. This is consistent with previous analyses, which found evidence of gene flow between wild and cultivated grapes in Western Europe, as well as between wild and domesticated apples (Myles et al., 2011; Cornille et al., 2012). Thus, it is worth noting that the distinction between cultivated crop and CWR, or progenitor species, in perennial crops is often blurred, as there may be shared segregating polymorphism and ongoing gene flow after domestication. Nevertheless, the notion of introgressing wild traits into elite germplasm is applicable across a diverse range of perennial crops, even those without a clear distinction between wild and cultivated species.

**FIGURE 2:**
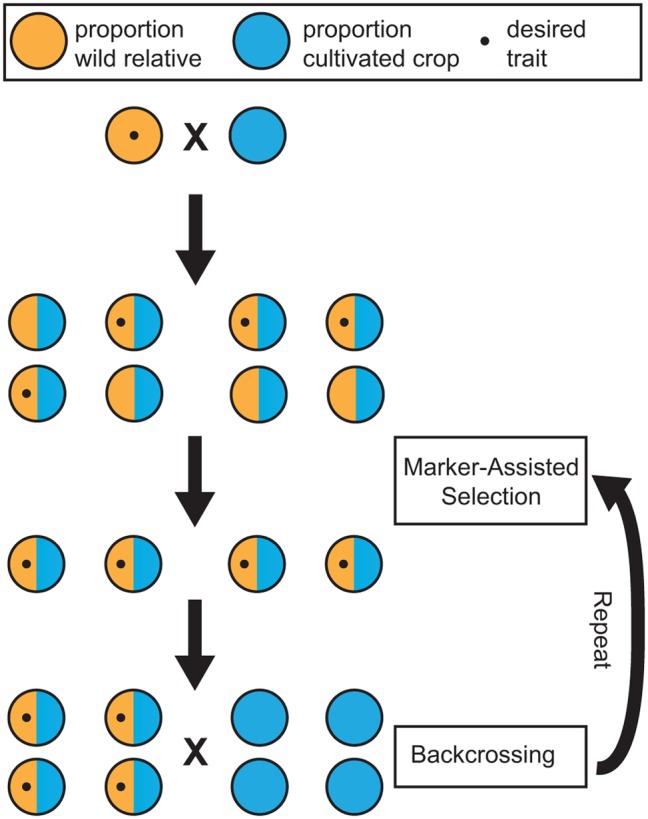
**Schematic of breeding using marker-assisted selection (MAS).** Wild relatives containing a trait of interest are crossed with a cultivated crop. In this example, the wild parent is heterozygous for a dominant Mendelian trait. With a marker associated with this trait, offspring can be screened for the trait and eliminated at the seedling stage. MAS ensures that the trait of interest is present in the progeny through several generations of backcrossing. Not shown here is that, with each generation, there is an increase in the proportion of cultivated ancestry while maintaining the desirable wild trait.

Marker-assisted selection (MAS) can increase the efficiency of incorporating desirable traits present in wild germplasm into domesticated, or elite, cultivars. MAS relies on genetic markers that are either causal for, or strongly linked to, a phenotype. The primary benefit of MAS is the ability to select individuals possessing a trait of interest at the seed or seedling stage using genetic markers. MAS allows the breeder to eliminate plants that do not possess the desired trait and may otherwise require a decade of cultivation to assess phenotypically. Instead, resources and space can be dedicated only to individuals with the desired characteristic. Plants with the desired trait can then be backcrossed to elite germplasm to maintain the wild trait of interest, while preserving important commercial traits (**Figure 2**).

Backcrossing to elite germplasm is crucial to ensuring traits of agricultural importance are maintained when breeding with wild relatives: the goal is to retain all desirable characteristics of the elite cultivars while introducing only the small number of desirable loci from the wild. However, a genomic assessment of wild ancestry in over 60 commercially grown hybrid grape cultivars found that one third had ancestry consistent with F1 hybridization. In fact, the study demonstrated that backcrosses to wild *Vitis* were more frequent than backcrosses to *V. vinifera*, indicating that repeated backcrossing to elite germplasm is not yet widely practiced (Migicovsky et al., 2016b). Breeding of perennial crops using wild relatives is still in its infancy. Through use of MAS and repeated backcrossing we can anticipate new, superior cultivars possessing useful traits from wild relatives while still maintaining the desirable characteristics of elite cultivars.

In addition to saving time, MAS can decrease the cost of perennial breeding using wild relatives. When compared to traditional fruit breeding, MAS was estimated to save up to 43% of operational costs over the first 6–8 years of an apple breeding program (Edge-Garza et al., 2015). MAS eliminates the need to phenotype and therefore offers the greatest cost and time savings for traits that may be difficult or expensive to measure, such as disease resistance, as well as traits expressed late in development, such as fruit quality (Töpfer et al., 2011). This review addresses the current use, future potential, and limitations of using wild germplasm for genomics-assisted breeding in perennial crops.

## Benefits: Disease Resistance

The majority of perennial crops are vegetatively propagated for decades, or even centuries, and are increasingly susceptible to evolving pathogens (Miller and Gross, 2011). In contrast, wild relatives have undergone natural selection in response to disease pressure and often harbor crucial resistance genes, which can be exploited through breeding. The monogenic nature of many resistance genes means MAS is especially feasible for introgression of disease resistance loci. Indeed, in a review of 19 different crops, over 80% of the traits incorporated from CWRs were involved in disease and pest resistance (Hajjar and Hodgkin, 2007). Similarly, another review of 104 MAS studies from 1995 to 2012 found 74% focused on disease and pest resistance (Brumlop et al., 2013). This demonstrates the widely acknowledged potential of improving crops through introgression of disease resistance traits from wild relatives.

The introgression of disease resistance from wild germplasm is perhaps best exemplified by modern grape breeders. While the genus *Vitis* contains over 60 inter-fertile species, approximately 99% of the world’s vineyards are planted with a single species, *V. vinifera* (This et al., 2006; Anderson and Aryal, 2013). While not commonly grown for commercial purposes, wild *Vitis* relatives possess many desirable traits not found within *V. vinifera*. For example, the effects of Pierce’s disease (PD) (*Xylella fastidiosa*) cost the California wine industry approximately $92 million annually (Alston et al., 2013). *Vitis arizonica*, a wild grape, is resistant to PD and has been used to develop PD-resistant wine grapes. MAS allows breeders to track PD resistance while backcrossing offspring repeatedly to *V. vinifera*. Breeding lines now possess PD resistance as well as 97% *V. vinifera* ancestry (Walker et al., 2014). Thus, MAS can facilitate the introgression of disease resistance from wild relatives while allowing for progeny that maintain desirable quality traits due to a high proportion of domesticated ancestry.

In addition to facilitating introgression of a single source of disease resistance, MAS is a valuable tool for introgression of several sources of resistance to the same disease, or even resistance to multiple diseases, through a process called pyramiding. For example, a *Muscadinia rotundifolia* ×*V. vinifera* cross was backcrossed four times to *V. vinifera*, resulting in the progeny ‘VHR 3082-1-42’ (Pauquet et al., 2001). ‘VHR 3082-1-42’ was then crossed with ‘Regent,’ a hybrid grape variety that is approximately 68% *V. vinifera*. The resulting progeny possess both powdery and downy mildew resistance genes from wild relatives as well as *V. vinifera* ancestry that likely exceeds 80% (Eibach et al., 2007; Migicovsky et al., 2016b). Wild grape species are also resistant to diseases such as black rot, crown gall, and others, all of which provide the opportunity for further improvement of commercial grape cultivars (Owens, 2008). The use of MAS to pyramid either several sources of resistance to a single disease, or resistance to multiple diseases, into a single cultivar is in its infancy. However, pyramiding of disease resistance markers promises to eventually result in grapes which require less chemical input to grow but still possess other commercially desirable traits.

There is also great potential for MAS in improving disease resistance in apple breeding programs. Apple scab (*Venturia inaequalis*) is one of the most destructive diseases in apple (*M. domestica*) and may require 20–30 fungicide treatments per season in commercial orchards. The wild relative *Malus floribunda* is widely used as a source of apple scab resistance. However, the resistance offered from *M. floribunda* is ineffective against certain strains of apple scab and a broader base of resistance is needed (Parisi et al., 1993; Soriano et al., 2009). Fortunately, resistance genes from several other wild relatives, including *Malus baccata jackii* (Gygax et al., 2004) and *Malus micromalus* (Patocchi et al., 2005), have been identified. Recent work used MAS to pyramid three scab resistance genes as well as genes for powdery mildew (*Podosphaera leucotricha*) resistance and enhanced fire blight (*Erwinia amylovora*) resistance into a single apple tree (Baumgartner et al., 2015). Many other desirable traits, including both abiotic and biotic stress resistance, are also found in wild *Malus* species (Volk et al., 2015). Thus, cultivars that contain ancestry from several wild relatives, each contributing desirable alleles to achieve the breeder’s target, are being developed. However, the achievements of breeding programs that have successfully exploited numerous wild perennial species are not yet widespread. The use of wild diversity will only increase in importance as pathogens continue to evolve.

In many instances, there is a great urgency to identify and exploit sources of disease resistance. In the case of banana, ‘Cavendish’ cultivars, were first grown due to their resistance to *Fusarium* wilt (*Fusarium oxysporum* f. sp. *cubense*) (Heslop-Harrison and Schwarzacher, 2007). Over 40% of bananas produced worldwide are ‘Cavendish’ cultivars and there are now reports of an evolved form of the pathogen to which it is susceptible (Hwang and Ko, 2004; Ploetz et al., 2007). Once infected with *Fusarium* wilt, the disease cannot be controlled and banana plants must be replaced with a new, resistant cultivar (Daly and Walduck, 2006). Resistant wild banana populations which co-evolved with the pathogen have been found and offer a valuable source of resistance to the newly evolved and highly pathogenic forms of *Fusarium* wilt (Javed et al., 2004). Recently, a marker for *Fusarium* wilt susceptibility with a discriminatory power of 93% was developed (Cunha et al., 2015). MAS is likely to facilitate the development of new resistant cultivars that will eventually replace the ‘Cavendish’ banana. It is possible for a single virulent strain to devastate an entire industry, and efforts to exploit wild relatives will become critical if the evolvability of pathogens is ignored.

The intense pressure to rapidly develop new, disease-resistant cultivars is not exclusive to the banana industry. Cacao (*Theobroma cacao*), used in the production of chocolate, is a perennial tree native to South America. As a result of disease outbreak in South and Central America over the past 200 years, 70% of the world production now occurs in Africa, 10% in Asia and only 20% in South America (Brown et al., 2005). Brazil, once the third largest producer of cacao, became a net importer of the crop following the arrival of *Moniliophthora perniciosa*, which causes Witches’ broom disease (Meinhardt et al., 2008). The use of a small number of cacao cultivars has left the crop vulnerable to disease and requires the continued expansion of production regions. However, pathogens continue to move to new cacao plantations. The only viable longterm solution in cacao, like banana, is the development of new, disease-resistant cultivars. Fortunately, wild populations of cacao still exist and have evolved in the presence of these pathogens. These wild relatives can be easily crossed with cultivated varieties, using molecular markers to accelerate the breeding process (Brown et al., 2005; Meinhardt et al., 2008; Zhang and Motilal, 2016). The recent evaluation of 520 wild cacao trees for important traits such as disease resistance, bean quality, and flavor will provide a valuable resource for future breeding (Zhang and Motilal, 2016). The cacao industry’s renewed focus on wild diversity serves as a warning to others who have yet to face the challenges that arise from evolving pathogen pressures. Only by establishing, maintaining, and evaluating diverse germplasm collections will the sources of pathogen resistance required in the future be readily available to breeders.

## Benefits: Fruit Quality

While most CWRs don’t taste very good, they may still possess unique fruit quality traits that can be incorporated into domesticated germplasm to create novel cultivars. Prior to the use of genomics, the fruity and aromatic “foxy” flavor found in the wild grape, *Vitis labrusca*, was introgressed into the domesticated grape, *V. vinifera*, for use in table grapes (Reisch et al., 2012). North Americans now commonly associate foxiness with “grape flavor,” especially in confectionary products. Although wild relatives are exploited primarily for their disease resistance, in some cases unique fruit characteristics possessed by wild relatives, but absent from cultivated germplasm, are targeted by breeders as well.

The appearance of a fruit, including color, is a critical breeding target in many fruit species. Most kiwifruits (*A. chinensis*) have green or yellow flesh, but red flesh is highly valued by consumers (Harker et al., 2007). The first red-fleshed commercial cultivar in the Chinese market, ‘Hongyang,’ required 20 years of breeding and selection to produce (Wang et al., 2002). Only a few red-fleshed kiwifruits have been collected for use in breeding. Wild kiwifruit with red flesh, including both *A. chinensis* and other *Actinidia* species, remain largely unexploited (Sui et al., 2013). Genomic work has begun in an effort to develop markers to easily identify red-fleshed kiwifruit. The identification of genetic markers for red flesh from wild relatives would allow breeders to select for this trait in kiwifruit, while minimizing the influence of any negative wild characteristics through repeated backcrossing (Wang et al., 2012). Fruit characteristics, such as color, are only visible in perennial crops after a juvenile phase and provide an excellent example of the potential for MAS to reduce the cost of breeding by allowing breeders to eliminate plants which do not possess the trait at an early stage. Reducing the cost of breeding through genomics can facilitate the development of more cultivars possessing unique fruit characteristics from wild relatives.

In addition to fruit appearance, improving nutritional qualities such as antioxidant capacity is an area of major interest, especially in raspberry (*Rubus idaeus*) and blackberry (*Rubus* spp.) breeding. A comparison between wild and cultivated raspberries found the highest antioxidant capacity in *Rubus caucasicus*, indicating the potential of increasing antioxidants in commercial cultivars through use of this species in breeding (Deighton et al., 2000). Similarly, work on blackberries found that wild genotypes had much higher levels of a key antioxidant than a commercial cultivar. Therefore, wild blackberries may be of use to breeding programs aiming to increase antioxidant content (Cuevas-Rodriguez et al., 2010). Raspberry and blackberry are just two examples of perennial crops which could benefit from breeding with wild relatives for desirable nutritional qualities, and it will be interesting to see how quickly—if at all—genomics-assisted approaches are adopted in these cases.

As evidenced by the examples provided in this review, MAS is incredibly useful for tracking traits that a breeder aims to introgress from wild relatives. However, in most cases, MAS will be used to maintain desirable traits from cultivated ancestors, rather than to introduce desirable quality traits from the wild. For example, in apple MAS is already in use for traits such as postharvest storability, firmness, acidity and skin color (Ru et al., 2015). When introgressing disease resistance from a wild relative, a breeder wants to retain only progeny with the desired fruit quality traits from the elite parent. Markers can be used to simultaneously track these desirable traits from the elite parent and the disease resistance from the wild parent. Thus, genomics is a valuable tool that enables breeders to efficiently select for the benefits offered by both wild and cultivated germplasm.

## Benefits: Rootstocks

A primary use of wild relatives in perennial breeding thus far has been for the development of rootstock varieties. Vegetatively propagated woody perennial crops are often shoots, or scions, grafted onto wild or hybrid rootstocks. Rootstocks can be used to improve perennial crops both above and below ground. Above ground, rootstocks can confer unique traits to the scion, such as precocity, or the reduction of time until a tree bears fruit, as well as the dwarfing of large trees. Below ground, targeted rootstock traits include drought tolerance, salt tolerance, and disease resistance (Warschefsky et al., 2016). While use of MAS in rootstock breeding has been limited to date, genomics can further improve rootstocks by facilitating the use of wild germplasm.

Given that most perennial crops are clonally propagated from a small number of elite cultivars, increased ease of travel and evolving pathogens pose a dangerous threat both to the scion as well as the portion of the plant found below ground. In the 1860s, the North American phylloxera louse (*Daktulosphaira vitifoliae*) devastated European vineyards. By attacking the roots of the plant, phylloxera kills *V. vinifera* vines within 1–2 years. Breeders used American wild *Vitis* species to develop resistant rootstocks, rescuing the wine industry, and *V. vinifera* wine cultivars are still grafted onto these rootstocks today (Alleweldt and Possingham, 1988; Zhang et al., 2009). Currently, bacterial canker (*Pseudomonas syringae* pv. *actinidiae*) poses a serious threat to kiwifruit worldwide. Fortunately, resistance to bacterial canker has been found in wild Chinese kiwifruit germplasm. A recent interspecies cross between wild *Actinidia eriantha* and cultivated *A. deliciosa* resulted in a rootstock resistant to bacterial canker. The same work discovered a genomic marker potentially useful for identifying bacterial-canker resistant hybrid rootstocks (Lei et al., 2014). As pathogens continue to evolve and spread, wild germplasm will be indispensable for use in rootstock breeding. Discovery of disease-resistant rootstocks using MAS can allow for the continued use of commercially successful scions while protecting the plant from diseases below ground.

Of the 25 most-produced fruit and nut crops, 20 may be grafted onto rootstocks, including grape and walnut (*Juglans regia*). The five crops not grafted are all monocots where grafting is not possible (Warschefsky et al., 2016). Given the global value of grafted perennial crops, the breeding of superior rootstocks is an area of great importance. While several generations of backcrossing may be necessary when crossing wild relatives with commercial scions to maintain fruit quality, wild trait introgression in rootstocks can be accomplished in fewer generations because the fruit quality of a rootstock cultivar is irrelevant. Use of wild relatives is further facilitated by graft compatibility between more distant relatives. For example, many stone fruits can be budded onto rootstocks developed for other *Prunus* species (Beckman and Lang, 2002). Peach (*Prunus persica*) and almond (*Prunus amygdalus*) × peach hybrid rootstocks with resistance to root-knot nematodes (*Meloidogyne* spp.) as well as adaptation to calcareous soil have been released. Both peaches and almonds, as well as some plum and apricot cultivars, can be grafted onto these rootstocks (Felipe, 2009). However, the most widely used rootstocks in almonds are still susceptible to lesion and ring nematodes, crown gall, and bacterial canker. The National Clonal Germplasm Repository (NCGR) of the United States Department of Agriculture Agricultural Research Service (USDA-ARS) in Davis, California is using almond relatives such as peach, wild almond species, and plums as potential donors for disease resistance and drought tolerance (Aradhya et al., 2015). The phenotypic evaluation of wild relatives, in combination with genomic data, enables the identification of markers linked to these desirable traits, allowing for donors to be efficiently selected. The development of disease resistant rootstocks through MAS using wild relatives is a topic of intense research interest in several perennial crops, and it is anticipated that this pursuit will result in substantial reductions in chemical input.

In addition to almond, the USDA-ARS is performing research on the use of wild relatives as potential sources of disease resistance for rootstocks in walnut. The primary walnut rootstock is ‘Paradox,’ a California black walnut *Juglans hindsii* × cultivated walnut *J. regia* hybrid which is tolerant of wet soil conditions, but susceptible to crown gall (*Agrobacterium tumefaciens*) (Hasey et al., 2013; Aradhya et al., 2015). Promising sources of disease resistance to crown gall and *Phytophthora* rots have been identified in wild species such as the North American black walnut (*J. hindsii*, *Juglans major*, and *Juglans microcarpa*) and Asian butternut species (*Juglans cathayensis* and *Juglans ailantifolia*). Mapping populations are currently being developed to identify disease resistance markers for MAS in walnut rootstocks (Aradhya et al., 2015). While molecular markers have not yet been used extensively in rootstock breeding, MAS can be used to screen hybrid progeny at a reduced cost and without the need to expose plants to pathogens in order to determine resistance status. Additionally, many of the traits important for rootstock breeding such as disease resistance, precocity, and dwarfing of the scion are targeted and defined. In comparison, in scion breeding far more complex traits, such as overall fruit quality, may be targeted. In turn, desirable rootstock traits are more likely to be controlled by a small number of genetic loci with large effects. Thus, the simple genetic architecture of most rootstock traits makes them amenable to genetic mapping as well as MAS. While wild relatives have long been viewed as a valuable tool for rootstock breeding, combining such benefits with genomics-assisted approaches is the crucial next step.

## Genomic Resources and Limitations: Mapping and Breeding

Despite the promise of wild relatives for improvement through MAS in perennial crops, there are several challenges to consider. In order to make use of wild relatives for MAS, the first step is to discover markers for traits of interest. Genome-wide association studies (GWAS) and linkage mapping are two methods used to establish genotype-phenotype relationships. GWAS relies on differences within a population of diverse, unrelated individuals in order to discover correlations between markers and traits. In comparison, linkage mapping exploits bi-parental crosses to map traits in the resulting progeny. One of the main advantages of GWAS over traditional linkage mapping is its superior mapping resolution. GWAS markers correlated with a phenotype are likely to be very close to the causal locus. In some cases, the likely causal genetic variant itself can be identified through GWAS (Migicovsky et al., 2016a). In linkage mapping, large genomic intervals, often spanning millions of nucleotides, are identified while the causal genetic variant is unlikely to be pinpointed. GWAS is particularly promising in perennials because of the time and cost required to generate bi-parental crosses. An additional benefit is that GWAS can be applied to germplasm collections that are already in the ground and waiting to be exploited (Chitwood et al., 2014). The discrepancy in mapping resolution between the two methods is a function of the number of recombination events captured by each method. In GWAS, a large number of unrelated individuals means that a large number of recombination events have occurred in the history of the genetic material being assessed. In linkage mapping, only the recombination events captured through the generation of the bi-parental cross can be exploited, resulting in relatively large chunks of DNA that share co-ancestry among individuals.

The high mapping resolution offered by GWAS is amplified in many perennials because of the relatively rapid linkage disequilibrium (LD) decay in high-diversity perennial crops. For example, LD decays within 200 bp in grape (Lijavetzky et al., 2007) and within 100 bp in apple (Migicovsky et al., 2016a) and Norway spruce (*Picea abies*) (Heuertz et al., 2006). This level of LD decay is far more rapid than in diverse populations of most well-studied annuals like rice (*Oryza sativa*, ∼75 to >500 kb; Mather et al., 2007), maize (*Zea mays*, 1–10 kb; Yan et al., 2009), and soybean (*Glycine max*, 336–574 kb; Hyten et al., 2007). The correlation between a marker and a causal variant is related to the level of LD between the two: the higher the LD, the more likely the marker will serve as an indicator for the presence of the causal variant. While rapid LD decay results in high mapping resolution, it also means that a very high density of markers is required for effective GWAS because the correlation among markers surrounding the causal variant decays so quickly. In some cases, generating sufficient coverage for GWAS by saturating the genome with markers may be prohibitively expensive due to rapid LD decay. However, the cost of marker discovery and genotyping is likely to continue to decrease, and it will therefore surely be feasible in the future for researchers to acquire the genotype data required for effective GWAS.

**FIGURE 3:**
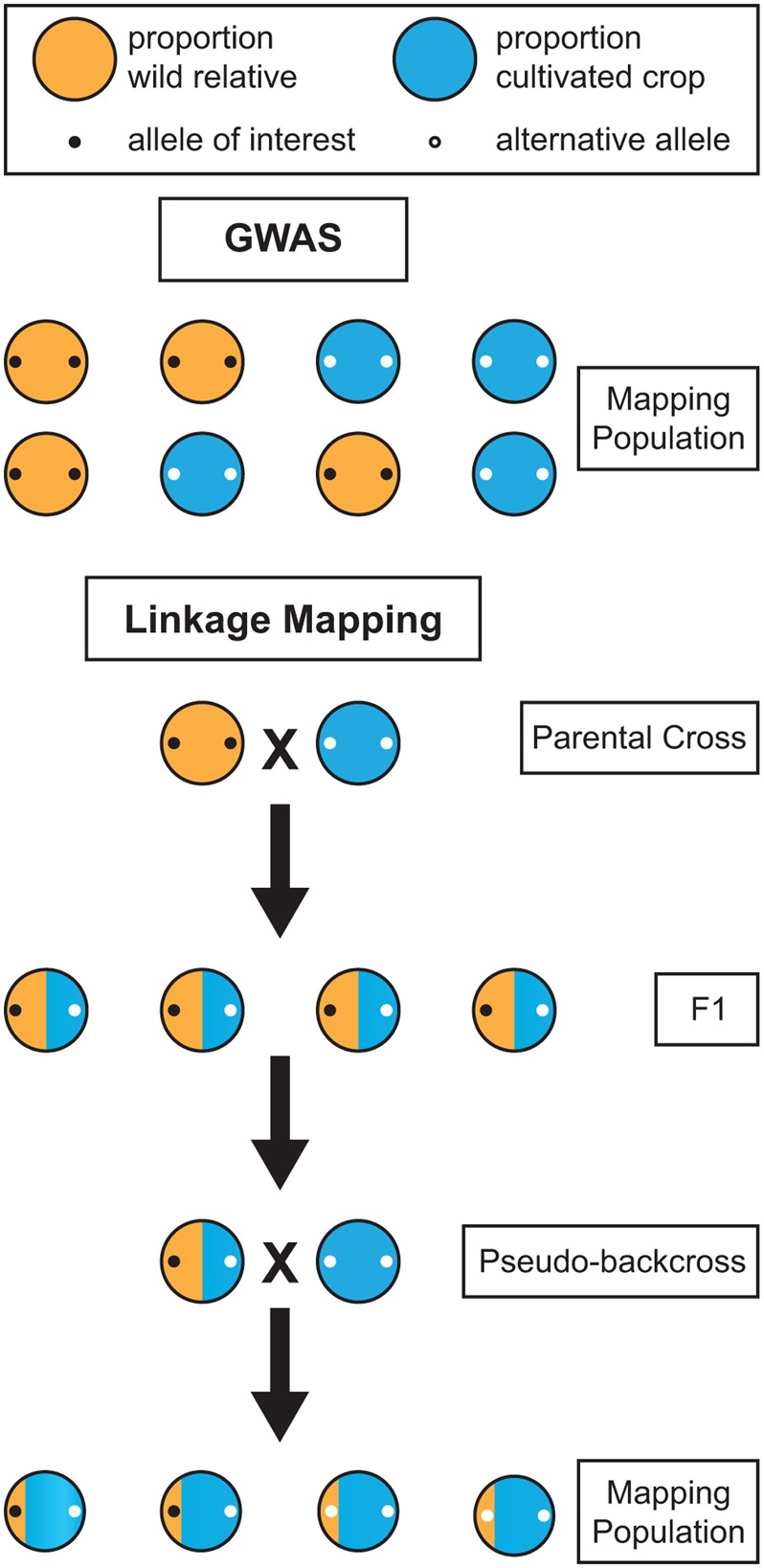
**Comparison of the effectiveness of genome-wide association studies (GWAS) and linkage mapping for mapping alleles of interest in wild relatives.** When an allele of interest is found only in wild germplasm it co-segregates with population structure and cannot be mapped using GWAS. Linkage mapping provides a viable alternative for mapping traits in wild relatives. However, in the F1 generation, alleles homozygous for alternative states in the wild and cultivated parent will not segregate. Thus, a backcross, or pseudo-backcross, is required to map most alleles of interest.

While GWAS in perennials is an attractive option, it is not always viable. Traits targeted by breeders are often present only within a wild relative species, and are completely absent within cultivated germplasm. Attempts to map such a trait in a population composed of the wild relative and the cultivated germplasm using GWAS would be futile because the trait co-segregates perfectly with ancestry. The marker you aim to uncover will be present in the wild relative but absent in the cultivated germplasm, but that is also the case for millions of other markers across the genome (**Figure 3**). When the phenotypes are perfectly segregated, GWAS is of no help and a bi-parental cross between the wild and cultivated populations must be made to genetically map the trait. Linkage mapping in the resulting bi-parental population allows for such co-segregating traits to be genetically mapped, because the confounding effects of population structure are broken through crossing. Thus, when mapping traits of interest found only in wild relatives, linkage mapping studies may be necessary due to co-segregation. However, it is sometimes the case that wild and domesticated germplasm share segregating polymorphism and are not significantly genetically differentiated, as is the case with apple and grape (**Figure 1**). In such instances, the confounding effects of co-ancestry may not be too severe and GWAS may be the genetic mapping option of choice. Additionally, when a phenotype is not perfectly co-segregated with ancestry, but rather differentially expressed in the two populations, it may be possible to perform GWAS using wild and domesticated plants. In this scenario, including both population structure and the SNP-by-population interaction in the GWAS model would help avoid false positives and ensure that SNPs are consistently associated with the trait across wild and domesticated populations (Biscarini et al., 2010). For each crop and phenotype of interest, the optimal genetic mapping approach, and the desired genetic composition of the population, will vary.

For co-segregating traits, linkage mapping provides a viable alternative to GWAS. In annual crops, it is typically performed through a cross of highly homozygous parents, often as a result of selfing. In perennials, the severe inbreeding depression and high level of heterozygosity require a mapping design in which parents are not selfed. As an alternative, the two-way pseudo-testcross design, in which two highly heterozygous parents are crossed, has been successfully applied in many perennials, beginning in 1994 with an interspecific *Eucalyptus* cross (Grattapaglia and Sederoff, 1994). However, the progeny resulting from a two-way pseudo-testcross will not segregate for markers homozygous for alternative alleles in the parental plants (**Figure 3**). Given that many wild traits of interest will likely fall into this category, mapping will require at least one generation of backcrossing before linkage mapping can be applied. However, many perennials also have high levels of inbreeding depression, so close relatives cannot be used when performing backcrosses. Instead, a cultivar that is not one of the parents from the initial cross should be used to perform pseudo-backcrossing. The combination of a two-way pseudo-testcross design and pseudo-backcrossing can enable the detection of markers for valuable traits in wild perennial relatives.

When introgressing regions of the genome associated with a phenotype, or quantitative trait locus (QTL), from wild germplasm, linkage drag may lead to undesirable phenotypes in the resulting progeny. Linkage drag is the result of unfavorable genes linked to a desirable QTL also being incorporated into the domesticated germplasm (Varshney et al., 2014). Additional generations of pseudo-backcrossing can reduce the effects of linkage drag. If undesirable loci are tightly linked to the locus of interest, it may be difficult to eliminate the impact of linkage drag through conventional breeding. Fine-mapping of a QTL can allow for the selection of individuals with specific recombination events that minimize linkage drag. Unfortunately, fine mapping requires generating a large number of crosses for sufficient recombination (Khan and Korban, 2012). Reduced recombination frequencies have also been reported surrounding loci introgressed for resistance from a related species, such as a 25-fold reduction in poplar (*Populus* spp.), providing further evidence that large populations will likely be needed for fine mapping (Stirling et al., 2001). As a result, the fine-mapping process is both expensive and time-consuming (Khan and Korban, 2012). Once a recombinant individual is identified, they can be used as a donor in breeding and backcrossing can continue for several generations using MAS.

In addition to eliminating linkage drag, fine-mapping may lead to the identification of causal alleles which can be subsequently incorporated into the genomes of domesticated crops through genetic modification (GM) or genome editing techniques. These techniques can be applied directly to the cultivar of interest, immediately incorporating the trait, and do not require multiple generations of backcrossing to eliminate linkage drag. This is especially valuable in perennial crops with a lengthy juvenile phase or infertile hybrid progeny. Previous work successfully generated transgenic bananas with resistance to *Fusarium* wilt, the major pathogen threatening banana production (Paul et al., 2011). Similarly, transgenic plantains (*Musa* spp.) with resistance to nematode pests *Radopholus similis* and *Helicotylenchus multicinctus* have been developed (Tripathi et al., 2015). In papaya (*Carica papaya*), the limiting production factor is the papaya ringspot virus (PRSV). While there have been attempts to transfer PRSV resistance from related wild *Vasconcellea* species to *C. papaya*, initially only F_1_ hybrids were possible as the resulting offspring were often infertile, preventing further backcrossing to *C. papaya* (Gonsalves et al., 2006). The first successfully backcrossed PRSV-resistant papaya was only reported in 2011, after 50 years of attempts (Siar et al., 2011). Instead, for almost two decades, papaya with transgenic resistance to PRSV have been cultivated in Hawaii (Gonsalves et al., 1998; Suzuki et al., 2007). Thus, GM is a valuable tool that can expedite the breeding of disease-resistant cultivars.

Currently, the most promising genome editing technique is CRISPR/Cas9, which is simple, flexible and efficient. CRISPR/Cas9 has been successfully employed in perennial species including apple (Nishitani et al., 2016) and sweet orange (*Citrus sinensis*) (Jia and Wang, 2014). Clearly, incorporation of desirable traits from wild relatives into perennial crops is not limited to MAS, but can also be achieved through GM. However, the social and regulatory acceptance of GM crops, including papaya outside of Hawaii, is often limited (Davidson, 2008). Acceptance of GM perennials is especially difficult since many are fruit crops that are consumed fresh. However, CRISPR/Cas9 may result in modified crops acceptable to those opposed to traditional GM techniques. For example, in 2015, Sweden confirmed that some plants edited using CRISPR/Cas9 were not considered genetically modified organisms (GMOs) under the European definition (Wolter and Puchta, 2017). One method for achieving further acceptance of crops modified using CRISPR/Cas9 is to avoid the use of foreign DNA, as recently achieved in maize (Svitashev et al., 2016) and bread wheat (*Triticum aestivum*) (Liang et al., 2017). Until global acceptance of CRISPR/Cas9 occurs, MAS continues to be a useful genomic tool for the introgression of desirable traits. Additionally, unlike genome editing, MAS remains useful when precise detection of causal loci is not possible and only markers highly correlated with the trait of interest are available.

The simple distinction between GWAS and linkage mapping is useful, but experimental designs that blur this distinction, and exploit the benefits of both methods, are uncovering numerous genotype-phenotype associations. For example, a Multi-parent Advanced Generation InterCross (MAGIC) population is created by intercrossing multiple parental lines rather than a single bi-parental cross. The increased level of recombination in the progeny allows for improved precision of mapping using inbred offspring (Cavanagh et al., 2008). In perennials, where the creation of inbred lines is often not possible, other designs have been implemented. For example, work in apple made use of a factorial mating design consisting of four female parents and two pollen parents (Kumar et al., 2012b). This family-based design allowed for the discovery of markers for traits such as fruit firmness, internal browning, and titratable acidity, which could be implemented in MAS (Kumar et al., 2013). Therefore, alternative mating designs are a promising tool for increased mapping resolution when performing linkage mapping between wild and domesticated crops.

The limited diversity—often a single bi-parental cross—exploited in traditional linkage mapping results in a mapping population where many QTL will not segregate and therefore not be detected. Further, due to a potentially small population size, small-effect QTL may not exceed the significance threshold. Significant markers identified are often only relevant to populations that share significant co-ancestry with the parents of the bi-parental mapping population. Thus, in comparison to GWAS, markers discovered using linkage mapping may not be predictive in diverse collections of germplasm (Owens, 2011). However, when identifying a marker for a trait from a wild relative, it is only necessary that the marker functions within that population, as a single source can be used as a donor for MAS. For example, while several sources of PD resistance have been used in grape breeding, the most important donor has been from a single *V. arizonica* accession, b43-17, which likely hybridized with *Vitis candicans* and is homozygous for monogenic resistance (Walker et al., 2014). Given that a single wild individual possessing a desirable trait is often sufficient for introgression into elite cultivars through MAS, transferability is of limited concern when exploiting alleles derived from a single wild relative.

A form of genomics-assisted breeding that is increasingly being used for complex traits is genomic selection (GS). GS is particularly useful when the breeder aims to predict a complex trait controlled by numerous QTL. In these cases, a small number of markers will not be sufficient for phenotype prediction. Many economically important traits, such as fruit quality, are polygenic and therefore controlled by a large number of loci. MAS uses specific molecular markers discovered through linkage mapping or GWAS. In comparison, GS uses all marker data as well as phenotype data from a population to predict a genomic estimated breeding value (GEBV) for an individual. Once a model has been validated, GEBVs can be calculated using only genotype information. However, while particular markers for MAS can be used to track a trait of interest across multiple generations, as breeding populations evolve, GS requires additional rounds of phenotyping in order to maintain an accurate prediction model (Varshney et al., 2014). Additionally, in contrast to MAS, GS requires genotyping a large number of markers, which may still be cost-prohibitive in many breeding programs. A combination of MAS and GS has been proposed in apple, in which monogenic traits are screened using MAS, followed by GS for complex traits. Such a strategy may benefit many perennial crops when introgressing multiple traits from wild relatives, especially to allow for durable disease resistance (Kumar et al., 2012a).

There are many tools and designs for genetic mapping and implementation of genomics-assisted breeding. The decision of which strategy to employ will vary depending on the genetic architecture of the trait as well as the genetic structure of the mapping and breeding populations. Similarly, the specific tool for introgression of markers is a complex decision that will require weighing factors such as the urgency of developing a new cultivar, the extent of linkage drag, and the acceptance of GM technology. While the optimal combination of genomic tools will differ by crop, the adoption of genomics-assisted breeding will ultimately enable breeders to efficiently and cost-effectively incorporate desirable traits that would otherwise remain locked away in wild germplasm.

## Genomic Resources and Limitations: Sequencing

Despite the immense potential of wild relatives for improving perennial crops, the first step to exploiting this resource through genomics-assisted breeding is discovering markers linked to useful phenotypes. While the genetic divergence between cultivated germplasm and wild relatives is precisely why wild relatives offer such unique and diverse traits, it may also cause difficulties for marker discovery and breeding. For example, when relatives differ in ploidy levels or total chromosome number, it may be difficult to produce fertile interspecific hybrids. The domesticated grape, *V. vinifera*, has 19 chromosomes while its relative, the American wild grape *Muscadinia rotundifolia*, has 20. However, progeny from *V. vinifera* ×*M. rotundifolia* have been generated and used for backcrossing to *V. vinifera.* Despite occasional sterility, successful pseudo-backcrossing occurred for six subsequent generations, allowing for the introgression of the *M. rotundifolia* gene for powdery mildew resistance, *Run1*, while maintaining a high proportion of *V. vinifera* (Bouquet et al., 2000). In cases of differing ploidy, one solution is the use of protoplast fusion, which has allowed for the creation of somatic hybrids in *Citrus* with ploidy differences as well as pollen/ovule sterility and abnormal chromosome pairing (Guo and Deng, 2001; Rauf et al., 2013). When fertile hybrids are still not possible and a causal locus has been identified, genome editing provides a viable alternative for introgression of valuable traits from wild germplasm.

In addition to the difficulties potentially associated with crossing more distant relatives, wild germplasm may have higher levels of diversity, and as such, DNA sequencing and genotyping tools designed for domesticated species may not function as successfully. For example, SNP arrays are widely used in humans, but do not function as well on organisms with greater genetic diversity because they are designed based on a reference genome. Insertion/deletion polymorphisms (InDels), copy number variants (CNVs), and presence-absence variants (PAVs) all reduce hybridization of a sample’s DNA to the probes on an array. Recent work in grape using the Vitis9KSNP array found 33–44% of genotype calls were discarded due to poor quality. In this case, hybridization intensities were more useful than genotype calls for genetic mapping precisely because of the probe-sequence hybridization issues caused by high levels of genetic divergence across grape species (Myles et al., 2015). Thus, when mapping in high diversity perennial crops with SNP arrays such as grape (Myles et al., 2010), peach (Verde et al., 2012), and apple (Bianco et al., 2016), use of hybridization intensities rather than genotype calls is a viable option to overcome the inevitably poor genotype quality.

As an alternative to a genotyping microarray, next-generation DNA sequencing technologies (NGS) such as restriction site associated DNA (RAD) sequencing (Baird et al., 2008) and genotyping-by-sequencing (GBS) (Elshire et al., 2011) do not require markers to be discovered prior to genotyping. The simultaneous discovery and genotyping of markers eliminates the need for DNA to hybridize to previously designed probes and makes NGS well-suited to high diversity species as well as wild relatives. However, in many cases, a reference genome is still used to map DNA sequence reads resulting from NGS and identify SNPs for association mapping or GS. Despite the proliferation of reference genome sequences, there is a lack of reference genomes for wild relatives. More than 100 plant genomes were sequenced between 2000 and 2014, but only 15 were wild relatives and over half of those were soybean (Michael and VanBuren, 2015). Thus, there is a clear need for reference genomes in wild relatives in order to map sequence reads allowing for the detection of SNPs for downstream analyses, ultimately allowing for genomics-assisted breeding.

While more genomic resources are still needed for wild species, the number of reference genomes available has continued to increase. Resequencing of several *Citrus* species including oranges, pummelos, and mandarins enabled researchers to determine the contributions of various wild progenitor species to cultivated citrus (Wu et al., 2014). Currently, a dozen wild *Prunus* species useful in hybrid breeding for rootstocks are undergoing genome resequencing by the USDA-ARS (Aradhya et al., 2015). Yet, in many cases, resequencing may not be sufficient for the detection of crucial genomic differences between wild and cultivated crops. Resequencing can detect SNPs as well as InDels when aligned to a reference genome. However, structural differences such as CNVs and PAVs are more difficult to detect. Within species, a large portion of the genome is present in only a subset of individuals. For example, transcriptome sequencing in maize was used to determine that only 16.4% of representative transcript assemblies were expressed in all 503 inbred lines examined (Hirsch et al., 2014). The divergence between wild relatives and cultivated plants is likely much greater. As a result, the genomic region of interest in a wild relative may be a sequence not present in the domesticated crop. DNA sequences present only in wild relatives require *de novo* assembly rather than resequencing to be mapped. The improvement of genomic resources, such as *de novo* assembly of wild relative reference genomes, can enable the discovery of markers for MAS and GS.

Finally, most sequencing results in some degree of missing data in the final table of genotypes. Missing sequence data can be filled in using imputation. However, imputation generally requires that genomic data be aligned to a reference genome. Popular imputation softwares, including Beagle (Browning and Browning, 2007) and fastPhase (Scheet and Stephens, 2006), rely on the input of SNPs ordered according to a reference genome, which is not possible for many wild relatives with limited genomic resources. Several methods such as Random Forest and *k*-nearest neighbors imputation (kNNI) can be used when a reference genome is not available (Nazzicari et al., 2016). LinkImpute is an imputation software based on kNNI, which updates the method to use linkage between markers rather than distance between samples when calculating neighbors. When compared to existing imputation methods, LinkImpute had a similar run time and accuracy to Beagle, despite not requiring positional information for markers (Money et al., 2015). As the ability to impute missing data without a reference genome improves, reduced representation sequencing techniques with high missing data, such as GBS, will continue to facilitate the discovery of new markers for genomics-assisted breeding in wild relatives.

While there is opportunity for great improvement to elite perennial crops through genomics-assisted introgression of traits from wild relatives, many barriers remain. Genomic tools designed for domesticated species are either not well-suited to more diverse wild relatives, or may be lacking completely. The same genetic divergence that has resulted in wild relatives harboring unique and desirable traits for breeding also results in difficulties in developing markers to introgress these traits into elite germplasm. However, given that DNA sequencing costs are likely to continue decreasing, it is essential that researchers begin planning for a future where the collection and analysis of DNA sequence data will not be the bottleneck to successful genetic mapping. Especially for perennial breeders used to working on timescales of decades, the focus should be on the collection of high-quality phenotype data that can always be paired later with genotype data as it becomes available. Now is the time to establish GWAS and linkage mapping populations that will enable powerful genetic mapping in a future where genotyping costs are negligible and the available genomic analysis tools are far superior to those available today.

## Further Limitations

Although the primary focus of this review is the use of genomics, it is worth noting that there are several difficulties unrelated to genomics that may limit the use of improvement using wild relatives. First, in order to make use of wild relatives for breeding, new germplasm must be collected. While some wild relative collections are well-characterized and actively in use, such as those described in this review, there are likely many benefits of wild germplasm that remain undiscovered. A focus on the collection and characterization of wild germplasm is the first step towards discovering which relatives and traits will be useful for breeding, and thus be exploitable through genomics.

Among the major barriers to improved characterization of wild germplasm are the locations where such germplasm may be found. Often, wild relatives must be collected from locations that are difficult to access, and thus collecting new wild germplasm can be an expensive and time-consuming process. For example, wild cacao is found in the tropical rainforests of South America (Lachenaud et al., 2007), while fruits and nuts may be expensive and difficult to retrieve from tall trees, and even vegetative samples may be bulky to transport (Aradhya et al., 2015). There are also compulsory quarantine requirements when transferring material between political boundaries. Several decades may pass between the collection of wild germplasm and their use by growers (Lachenaud et al., 2007). Finally, it is important to consider the cultural and financial ramifications of collecting wild relatives. In the past, germplasm has been collected from farmers and communities without compensation or recognition. In such a scenario, seeds may be taken from one country and used to benefit the private sector in another country. While there is ample opportunity for commercial crops to benefit from wild relatives, it is necessary that farmers and communities which have preserved wild relatives receive adequate credit and compensation for use of such resources (Montenegro, 2016).

The introgression of valuable wild traits into domesticated crops can only occur when breeders have access to these relatives through gene banks. The collection of new samples for marker discovery poses a major limitation to establishing such collections. Wild relatives are very under-represented in gene bank collections. A recent overview of over 1,000 taxa in 81 crops found that no CWR germplasm existed in gene banks for 29% of taxa, while 24% had fewer than 10 accessions. Over 95% of taxa had insufficient wild relative representation in gene banks, clearly supporting the need for better collection of wild germplasm, in order to make use of it in breeding (Castañeda-Álvarez et al., 2016). Future collection of germplasm is also threatened due to habitat destruction and climate change (Maxted et al., 2012). As the power of genomic tools increases, genomics will become increasingly effective for introgression of wild traits into perennial crops. However, the ability to exploit wild relatives for breeding requires that this diversity be protected for future use through gene banks and habitat conservation. Preservation of wild relatives will require a complex approach across many environments on a local, national and international scale (Montenegro, 2016). It is crucial to begin exhaustive sampling and extensive evaluation of wild germplasm for all major perennial crops, an enormously expensive and time-consuming undertaking. However, such projects are essential to ensuring a safe and secure future food supply as clonally propagated cultivars continue to be threatened by a constantly evolving environment.

## Future Directions

An essential step toward the adoption of genomic markers from wild relatives will be methods that accelerate the juvenile period in order to increase the efficiency of backcrossing progeny to domesticated germplasm. While the use of genomics-assisted breeding can increase the efficiency of selecting for traits of interest and decrease the number of plants that must be propagated, the long juvenile period of many perennials still poses a constraint on the rate of crop improvement.

A solution to the problem of long juvenile periods has been found in grapes. In grapes, ‘microvines’ possessing a *Vvgai1* mutant allele display dwarfism, a short generation time and continuous flowering. In comparison to the 2–5 years of juvenility generally required for grapes, the *Vvgai1* mutant produces fruit 2 months after germination. In addition to allowing for the rapid cycling of generations, microvines take up less space and could be a valuable tool for genomics studies and MAS (Chaïb et al., 2010). For example, recent work used microvines to aide in QTL identification for traits such as berry acidity (Houel et al., 2015). In apple, an early flowering transgenic line containing the *BpMADS4* gene from silver birch (*Betula pendula*) was combined with MAS to pyramid resistance to apple scab, powdery mildew, and fire blight (Flachowsky et al., 2011). However, while transgenic lines are incredibly helpful for decreasing the generation time while breeding, it is often desirable to have a final cultivar for release that does not contain the transgene and is not considered a GMO. This scenario is facilitated by a transgene that is dominant and heterozygous, resulting in only 50% of offspring possessing the gene in each generation. Thus, once the rapid cycling of generations is completed, a non-GMO tree possessing desirable traits from wild relatives—but not the transgene—can easily be selected (Flachowsky et al., 2011). The creation of similar mutants in other species, which reduce the juvenile phase in long-lived perennials, will be essential to the efficient application of MAS.

As an alternative to transgenics, virus-induced gene silencing (VIGS) can also be used to shorten the juvenile phase in perennials. VIGS uses a viral vector to infect a plant with a particular gene, resulting in an RNA-mediated defense which silences expression of the gene within the plant (Lu et al., 2003). The *apple latent spherical virus* (ALSV) does not induce disease symptoms in the infected plant and can be used as a vector for VIGS (Igarashi et al., 2009). When ALSV is used to express *Arabidopsis thaliana* florigen while silencing expression of *MdTFL1-1* in apple or *PcTFL1-1* in pear, flowering time can be reduced to 3 months or less. As genes involved in flowering are identified in other perennials, VIGS could be used to silence these genes and thus shorten the juvenile period (Yamagishi et al., 2011, 2016). ALSV has several other valuable characteristics which make it attractive for use in breeding. The virus was not detected in neighboring trees in an orchard where it had been present since 1984, suggesting there was no vector for transmission present in the sampled orchard and horizontal transmission via pollen did not occur (Nakamura et al., 2011). Additionally, approximately 99% of seedlings from ALSV-infected trees can be considered virus-free (Kishigami et al., 2014). Finally, ALSV can be eliminated from an infected tree using high temperature, allowing for vegetative propagation of that tree and resulting in fruit exempt from restrictions on GMOs (Yamagishi et al., 2016). Therefore, VIGS is a promising method for reducing the juvenile phase in perennials, allowing for a shorter generation time and thus facilitating backcrossing when breeding with wild relatives.

The ability to genotype plants using MAS at the earliest stage of development will allow for the least amount of time and resources to be spent propagating plants which do not carry the marker of interest. While extraction of DNA from seeds is possible for several plants, in perennials it is generally required that plants germinate in order to collect DNA from leaf tissue. Many tree fruits and nuts require a seed dormancy period of up to 12 weeks at low temperatures prior to germination. The development and improvement of methods which overcome seed dormancy could decrease the time prior to genotyping and the generation time between crosses. Several techniques for overcoming seed dormancy include the dissection of embryos and application of bioactive gibberellins or nitric oxide (van Nocker and Gardiner, 2014). Work describing the non-destructive ability to extract DNA from seeds, although recently published in soybean, has been limited so far (Al-Amery et al., 2016). In such a scenario, only the seeds with the desired trait would be germinated, greatly improving the efficiency and decreasing the cost of each breeding cycle.

To facilitate DNA sequence mapping and marker discovery for wild relatives, improvement of genomic resources is needed. As such, there is an urgent need for reference genomes in wild species, or the development of pan-genome sequences that include sequence from both wild and domesticated relatives. To characterize the pan-genome of poplar, recent work performed genome-wide analysis of structural variation in three intercrossable poplar species (Pinosio et al., 2016). Similar efforts are required in most other perennial species. Resequencing of wild germplasm, in combination with *de novo* assembly, will not only improve our understanding of the domestication history of perennial crops, but also enable the genetic mapping of important traits that can be used for genomics-assisted breeding.

While this review focuses on the potential of genomics-assisted breeding, and in particular MAS, it is worth noting that these tools will always be used in combination with traditional evaluation of cultivars when selecting new varieties. Breeders will always grow and evaluate plants prior to commercial release, but genomics can speed up reaching that final evaluation. Moreover, there are certainly cases where MAS may not even be desirable. For example, when selecting for red fruit flesh in apple, the same anthocyanin-regulating transcription factor often leads to red foliage and therefore trees with this trait can be easily identified before fruit production (Chagne et al., 2007; Espley et al., 2009). However, there is also a paralogous gene for red fruit flesh color where red foliage does not occur and MAS could be valuable in those instances (Chagne et al., 2013). Due to the cost and labor expense of MAS, previous work selecting for downy and powdery mildew resistance in grape included both phenotypic and marker-assisted selection. The initial population of interest consisted of 119 plants inoculated with downy mildew. Seedlings resistant to downy mildew were then screened for powdery mildew resistance. Finally, the 20 seedlings resistant to both diseases were tested using MAS, resulting in a final reduction to only four seedlings (Eibach et al., 2007). In this case, while phenotype selection was effective, MAS allowed for an improved reduction in the number of seedlings. When applying MAS to perennial crops, the greatest cost-savings will occur if testing occurs at the seed or seedling stage. MAS is particularly useful for traits that are difficult, expensive, or time-consuming to phenotype, such as fruit traits and disease resistance. To be of use, the markers must be economical to discover as well as test. Lastly, MAS requires a robust marker-trait association which improves the breeder’s ability to select for individuals possessing a particular trait. Thus, while low cost MAS can facilitate the introgression of specific traits of interest from wild relatives, it will ultimately only be useful when the cost of phenotyping is higher than the cost of discovering markers and genotyping (Luby and Shaw, 2001).

Lastly, a major barrier to more widespread adoption of MAS is often not the lack of genomic resources for wild relatives or the cost of genotyping, but the ‘phenotyping bottleneck’ present when characterizing germplasm. While the cost and speed of collecting genomic data has continued to decrease, phenotyping remains slow and expensive (Burleigh et al., 2013). Given that high-quality phenotype data is required for well-powered QTL analyses, the improvement of phenotyping technologies is a major area of current research interest. The development of new, high-throughput (HT) phenotyping technologies has begun, including advances in image analysis and robotics (Furbank and Tester, 2011). Improvement to phenotyping technologies will aid in the characterization of wild germplasm, a task which is particularly challenging due to the high level of diversity present. Thus far, HT phenotyping technologies have focused on annual crops such as rice (Tanger et al., 2017) and cotton (*Gossypium barbadense*) (Andrade-Sanchez et al., 2014), neglecting diverse perennial crops. One example of a technology useful for wild relatives is Field Book, an open-source application for collecting field data that eliminates the need to transcribe handwritten notes (Rife and Poland, 2014). As phenotyping technology for perennial crops and wild relatives improves, so will the ability to detect markers which can be exploited for genomics-assisted breeding. Thus, phenotyping of wild relatives, while expensive, is a necessary task. Additionally, good quality phenotype data will continue to have value in the future. Phenotype data can be collected now but analyzed in the future when, for example, the cost of whole genome sequencing is no longer prohibitive.

Ultimately, although genomics-assisted breeding has been used to introgress traits from wild relatives into perennial crops in the past, there are still many areas in which future work is required to improve this process. The use of genomic tools such as those which reduce the generation time for long-lived perennial crops and allow for DNA extraction from seeds—and the continued development of such tools—are two crucial steps in facilitating the use of MAS in perennials. To make use of markers in breeding, they must first be discovered, and as such improvement to genetic mapping techniques and resources will be necessary. Finally, MAS is especially valuable for the introgression of multiple traits as well as those that are difficult or expensive to phenotype. However, the usefulness of MAS relies on the ability to discover and genotype markers for less than the cost of phenotyping all progeny. As technology improves and the cost of marker discovery decreases, it will become increasingly feasible to introgress useful traits from wild relatives into elite perennial cultivars, resulting in the much-needed improvement of crops that may have been clonally propagated for centuries.

## Conclusion

There are clearly many traits such as disease resistance, fruit quality, and rootstock characteristics which would benefit domesticated perennials but are locked in undesirable, wild germplasm. Use of MAS can enable breeders to unlock the potential of wild germplasm by facilitating selection at an early stage of development—or even as a seed—allowing for less time and money to be spent growing plants which will inevitably be discarded. However, when crossing wild relatives and elite cultivars there are certain limitations and difficulties. Often many generations of backcrossing are required to decrease linkage drag and other wild characteristics. Use of GM technology can help reduce the amount of time required for breeding, but decades may still be required for consumer and regulatory acceptance. The development of CRISPR/Cas9 for genome-editing is a promising alternative to traditional methods. Both MAS and genome editing require the initial discovery of markers, which is complicated by the fact that alleles for traits of interest often co-segregate with millions of other alleles in wild germplasm. Yet, the potential benefit of accessing unique and desirable traits in wild germplasm could revolutionize perennial crop improvement. Unfortunately, the discovery of useful markers using GWAS and linkage mapping may still require decades to yield results. Thus, it is essential the collection and characterization of wild relatives begin immediately, while genomic and phenomic tools suited to diverse germplasm continue to improve. The continued vegetative propagation of domesticated perennial cultivars affords pathogens the opportunity to become increasingly effective while robbing both growers and consumers of the unique and desirable traits present in wild germplasm. After decades, or even millennia, of growing the same perennial cultivars frozen in genetic time, the decreasing costs of sequencing can finally allow us to harvest the potential of wild relatives through genomics-assisted breeding. We have only begun to enjoy the benefits of wild relatives in perennial crop improvement, and continued technological advances will surely result in the more efficient development of tastier food that requires less chemical input to grow.

## Author Contributions

All authors listed have made substantial, direct, and intellectual contribution to the work, and have approved it for publication.

## Conflict of Interest Statement

The authors declare that the research was conducted in the absence of any commercial or financial relationships that could be construed as a potential conflict of interest.
